# Bone Mineral Density Changes in Relation to Environmental PCB Exposure

**DOI:** 10.1289/ehp.11107

**Published:** 2008-05-16

**Authors:** Susan Hodgson, Laura Thomas, Elena Fattore, P. Monica Lind, Tobias Alfven, Lennart Hellström, Helen Håkansson, Grazia Carubelli, Roberto Fanelli, Lars Jarup

**Affiliations:** 1 Institute of Health and Society, Newcastle University, Newcastle upon Tyne, United Kingdom; 2 Department of Epidemiology and Public Health, Imperial College London, United Kingdom; 3 Department of Environmental Health Sciences, “Mario Negri” Institute for Pharmacological Research, Milano, Italy; 4 Institute of Environmental Medicine, Karolinska Institutet, Stockholm, Sweden; 5 Department of Environmental Medicine and Public Health, County Council of Kalmar, Oskarshamn, Sweden; 6 Division of Occupational and Environmental Medicine, Department of Molecular and Clinical Medicine, Faculty of Health Sciences, Linköping University, Linköping, Sweden

**Keywords:** bone mineral density, *p,p′*-DDE, polychlorinated biphenyls, toxic equivalents

## Abstract

**Background:**

Bone toxicity has been linked to organochlorine exposure following a few notable poisoning incidents, but epidemiologic studies in populations with environmental organochlorine exposure have yielded inconsistent results.

**Objectives:**

The aim of this study was to investigate whether organochlorine exposure was associated with bone mineral density (BMD) in a population 60–81 years of age (154 males, 167 females) living near the Baltic coast, close to a river contaminated by polychlorinated biphenyls (PCBs).

**Methods:**

We measured forearm BMD in participants using dual energy X-ray absorptiometry; and we assessed low BMD using age- and sex-standardized *Z*-scores. We analyzed blood samples for five dioxin-like PCBs, the three most abundant non-dioxin-like PCBs, and *p,p*′-dichloro-phenyldichloroethylene (*p,p*′-DDE).

**Results:**

In males, dioxin-like chlorobiphenyl (CB)-118 was negatively associated with BMD; the odds ratio for low BMD (*Z*-score less than −1) was 1.06 (95% confidence interval, 1.01–1.12) per 10 pg/mL CB-118. The sum of the three most abundant non-dioxin-like PCBs was positively associated with BMD, but not with a decreased risk of low BMD. In females, CB-118 was positively associated with BMD, but this congener did not influence the risk of low BMD in women.

**Conclusions:**

Environmental organochlorine exposures experienced by this population sample since the 1930s in Sweden may have been sufficient to result in sex-specific changes in BMD.

It is well known that hormones, vitamins, pharmaceuticals, and metals can have adverse effects on bone. Bone effects, mainly congenital, have also been linked to persistent organochlorine exposure following a few notable poisoning incidents. High-level, accidental dietary exposure to hexachlorobenzene resulted in severe osteoporosis (Cripps et al. 1984; Gocmen et al. 1989), and infants exposed *in utero* to high concentrations of polychlorinated biphenyls (PCBs) and poly-chlorinated dibenzofurans developed irregular calcification of their skull bones (Miller 1985).

Recent epidemiologic studies in populations with environmental organochlorine exposure have yielded inconsistent results regarding whether organochlorine exposure has an effect on bone properties. Several single-sex studies have indicated a possible association between organochlorine exposure and bone quality. In 115 Swedish men from the general population, a significant association was seen between serum concentrations of the organochlorines chlorobiphenyl (CB)-28 and γ-hexachlorocyclohexane and broadband ultrasound attenuation [an indirect estimation of bone mineral density (BMD)] of the left os calcis, and between CB-167 and total-body BMD after adjustment for confounding variables (Glynn et al. 2000a). In 68 sedentary Australian women, a negative correlation between *p,p*′-dichlorodiphenyldichloroethylene (*p,p*′-DDE) levels and BMD was observed, suggesting that past exposure to DDT may be associated with reduced BMD in women (Beard et al. 2000). Other studies have not found organochlorine exposure to be associated with bone effects. In 103 peri- and post-menopausal women from the United States, no correlation was found between *p,p*′-DDE concentration and bone density or rate of bone loss at the spine or radius (Bohannon et al. 2000). In 153 peri- and postmenopausal Inuit women with high organochlorine exposure from their seafood diet, concentrations of CB-153 were inversely correlated with bone parameters in univariate analyses, but, again, this relationship was no longer evident after adjustment for potential confounding variables (Cote et al. 2006).

One study investigated bone effects in males and females from Sweden. In the initial study, the risk of hospitalization from fracture in fishermen and their wives was compared between those living on the east coast of Sweden (near the Baltic sea, where environmental organochlorine exposure is likely attributable to consumption of contaminated fatty fish) and those living on the west coast (Alveblom et al. 2003). There was a significantly increased incidence rate ratio for vertebral fractures among east coast fisherman’s wives, with a similar (nonsignificant) tendency in east coast fishermen after adjustment for age and calendar year, compared with those dwelling on the west coast. It should be noted that this exposure assessment is rather crude and that other fracture types did not show a similar trend. Following from this study, the relationship between serum levels of CB-153 and *p,p*′-DDE and BMD in a subset of the Baltic coast fishermen (*n* = 196) and their wives (*n* = 184) was further studied (Wallin et al. 2005). Univariate analyses revealed significant negative associations between CB-153 and BMD in males and females, but after adjustment for age and body mass index (BMI), these relationships did not persist.

Although the epidemiologic evidence is inconsistent, an increasing number of experimental studies lend biological plausibility to organochlorine-induced bone effects (Andrews 1989; Badraoui et al. 2007). Animal studies also have demonstrated that the timing of exposure can be critical, with effects being observed at lower dose levels when exposure occurs during earlier life stages; furthermore, the same compound seems to have the potential to modulate bone quality differently depending on the developmental stage at exposure (Jamsa et al. 2001; Miettinen et al. 2005). The estrogen status of the exposed individual has also been shown to influence the toxicity of organochlorines on bone. Studies in rats exposed to the dioxin-like CB-126 showed that bone strength and composition are impaired and that estrogen can modulate the induced effects depending on the estrogen status of the individual (Lind et al. 1999, 2000, 2004). CB-126 exposure did not affect bone mineral density or trabecular bone volume of tibia in sham-operated rats. In contrast, in estrogen-deprived ovariectomized rats, CB-126 exposure resulted in a decreased length and increased bone mineral density of tibia. Furthermore, estrogen supplementation modulated CB-126-induced effects on bone tissue in rats (Lind et al. 1999, 2000, 2004). Because of these various influences, the effect of exposure to a number of different organochlorines is likely to be difficult to predict; nonetheless, these animal studies suggest a possible causal relationship between organochlorine exposure and adverse bone effects. Such a relationship, if applicable to humans, could play a role in the observed increase in osteoporosis and osteoporotic fractures in the western world (Genant et al. 1999; Ismail et al. 2002).

The purpose of the present study was to investigate the relationship between organo-chlorine exposure and BMD in a subset of the Osteoporosis Cadmium as Risk Factor (OSCAR) cohort. The OSCAR cohort was established to investigate the effect of low-level cadmium exposure on bone and kidneys (Alfven et al. 2002, 2004). Because many of the examined individuals live close to the Baltic coast, these people might also have experienced elevated dietary PCB exposure from consuming contaminated fatty fish. In addition, there was potential for PCB exposure from a nearby contaminated river, which was polluted with PCB containing paper pulp from an upstream paper mill (Bremle et al. 1998).

## Materials and Methods

Full details of the OSCAR study population can be found elsewhere (Alfven et al. 2002). In brief, subjects 16–80 years of age who had resided near a nickel-cadmium battery plant for at least 5 years between 1910 and 1992 were invited to participate in the OSCAR study; 904 (62%) agreed to participate. In addition, workers with previous or current occupational exposure were invited to participate, and 117 (48%) agreed to take part. In total, 1,021 individuals (60%) agreed to participate in the study. The OSCAR participants provided information on employment, residence, smoking, diet, medical history (especially regarding kidney diseases and diseases related to osteoporosis). Specially trained nurses collected urine and blood samples and measured height and weight. Forearm BMD was measured with an ambulant instrument (Osteometer DTX-200; Meditech A/S, Rødovre, Denmark), using dual energy X-ray absorptiometry, which is commonly used to evaluate BMD (Alfven et al. 2000; Glynn et al. 2000a; Wallin et al. 2005). The distal site in the nondominant forearm was measured with the patient in a supine position. This site includes both the radius and the ulna from the 8-mm point (the point where the radius and ulna are separated by 8 mm) and 24 mm proximally, and contains 10–20% trabecular bone (Schlenker and VonSeggen 1976). Internal variation was checked by daily calibration using a phantom, and the measurements from the ambulant instrument were validated against a stationary hospital-based instrument (Jarup et al. 1998).

In addition to investigating BMD as a continuous variable, we created a dichotomous variable representing presence or absence of low BMD for use in logistic regression analyses. Low BMD was assessed by computing an age- and sex-standardized *Z*-score. A common definition of low BMD is *Z*-score less than −1 (Kanis et al. 1997), which indicates 1 standard deviation below a sex- and age-standardized mean obtained from a reference population provided by the instrument supplier.

Blood samples from a subset of the OSCAR cohort consisting of participants ≥ ^60^ years of age (*n* = 325) were included in this study [insufficient sample remained and/or labeling was unclear in 23 out of 348 samples (6.6%), meaning that we could assess only 325 out of 348 eligible participants]. Samples were analyzed for five mono-*ortho* chlorine substituted congeners (CBs 105, 118, 156, 157, and 167), expressed in terms of toxic equivalency (TEQ_mono-_*_ortho_*), and individual CB-118 levels, to assess the effect of the dioxin-like activity. The sum of three most abundant non-dioxin-like (or di-*ortho* chlorine substituted) CBs 138, 153, and 180 (Σ_3PCB_), and individual CB-153 levels were also analyzed. We measured CB-153 because the concentration of this congener correlates well with total and dioxin-like PCB concentrations in plasma and serum (Glynn et al. 2000b). Σ_3PCB_ is considered to be an indicator of the content of total PCBs in human samples; this measure represented, on average, 61% of the human PCB body burden (European Food Safety Authority 2005). Finally, we also analyzed *p,p*′-DDE, the persistent metabolite of DDT (dichlorodiphenyltrichloroethane) because this organochlorine has been measured in other studies exploring the effects of organochlorines on BMD (Beard et al. 2000; Glynn et al. 2000a; Wallin et al. 2005). Ethical approval for this study was obtained from the Karolinska Institutet ethics committee, and all participants gave their written, informed consent before the OSCAR study.

The analytical method for the measurement of PCBs was initially developed in serum (Mariani et al. 2002; Turci et al. 2004), but has been extended to plasma and whole blood. Before the analysis of the OSCAR samples for this study, analyses of PCBs in whole blood, serum, and plasma derived from the same blood sample were performed, and results showed no major differences between these matrices in the distribution of the congeners, and the coefficients were close to 1 [see Supplemental Material, Figure 1 (online at http://www.ehponline.org/members/2008/11107/suppl.pdf)]. Analysis of whole blood, however, has been suggested to better reflect the PCB body burden (Janak et al. 1999). Briefly, after thawing, aliquots of 0.5 mL of blood samples were spiked with a mixture of the eight PCB congeners (^13^C_12_ labeled; Wellington Laboratories, Guelph, Ontario, Canada) and *p,p*′-DDE. Sulfuric acid was added to a final volume of 2.5 mL. After cleanup overnight on an Extrelut column (Merck, Darmstadt, Germany), samples were concentrated to a small volume and analyzed by high-resolution gas chromatography (CG)–high resolution mass spectrometry using a trace CG (Finnigan, Bremen, Germany) provided with a GC PAL autosampler (CTC-Analytics, Zwingen, Switzerland) coupled to a Thermofinnigan MAT95XP mass spectrometer. The mass spectrometer was operated in electron ionization mode, using selected ion monitoring at a mass resolution of 10,000. Quantification was done by the isotopic dilution method. We carried out blank analyses routinely for every 20 samples, and for the definition of the limit of detection (LOD), a signal-to-noise ratio of 3:1 was chosen. For the TEQ_mono-_*_ortho_* calculation the latest toxic equivalency factors (TEFs) were used (Van den Berg et al. 2006) and when the CB concentration values were below the LOD, they were entered as 0 (lower bound method).

We evaluated associations between organochlorine levels and BMD in SPSS 12.0 for Windows (SPSS Inc., Chicago, IL, USA) using univariate and multivariate linear regression and logistic regression analyses. Given the probable hormonal action of organochlorines on bone, and given the very large difference in osteoporosis incidence in males and females after 60 years of age, it seemed most appropriate to assess the relationships between organo-chlorines and BMD measures in males and females separately. Information on potentially confounding lifestyle factors and food consumption habits collected for the OSCAR cohort were included in the linear regression model if they fulfilled the stepwise criteria (probability-of-*F*-to-enter ≤ 0.10, probability-of-*F*-to-remove ≥ 0.15) to aid the interpretation of the results. Because the OSCAR cohort was originally set up to explore the effect of cadmium on BMD, blood cadmium was included as a potential confounding variable in the linear and logistic regression analyses presented.

## Results

The blood concentration levels ranged from 0.002 to 0.067 pg TEQ_mono-_*_ortho_*/mL for the dioxin-like PCBs, from 438 to 8,960 pg/mL for Σ_3PCB_ and from 16.4 to 17,500 pg/mL for *p,p*′-DDE (Table 1). Women had significantly higher levels of CB-118 and *p,p*′-DDE than did men.

In unadjusted analyses, significant negative correlations were seen between CB-118 and BMD (Pearson correlation coefficient = −0.130; *p* = 0.020) when males and females were investigated as one group. When males and females were investigated separately, none of the individual CB congeners was significantly correlated with BMD. There were significant positive correlations between each of the five organochlorine markers of interest. The dioxin-like organochlorines CBs 105, 118, 156, and 167 were all strongly correlated with TEQ_mono-_*_ortho_* in males, females, and the whole group (Pearson correlation coefficient > 0.6); CB-157 showed far weaker, even non-significant correlations with the other organo-chlorines. CB-118 showed consistently strong correlations with the TEQ_mono-_*_ortho_* measure (correlation coefficient > 0.9) and with the Σ_3PCB_ measure (correlation coefficient > 0.6). The abundant non-dioxin-like congeners were always strongly correlated with each other (correlation coefficient > 0.6), and CB-153 was always very strongly correlated with the Σ_3PCB_ measure (correlation coefficient > 0.9). In males, *p,p*′-DDE showed relatively strong correlations with TEQ_mono-_*_ortho_*, CB-118, Σ_3PCB_, and CB153 (correlation coefficient ~ 0.5), though these correlations were weaker in women (correlation ~ 0.4).

As anticipated, in bivariate analyses, age was negatively correlated with BMD, and BMI was positively correlated with BMD (data not shown). In males, alcohol was negatively correlated and milk consumption was positively correlated with BMD. In females, age at menopause and number of reproductive years (age at menopause minus age at menstruation) were both positively correlated with BMD; age at menstruation was negatively correlated with BMD. Contraceptive pill use was associated with an increased BMD, as was ever having been pregnant.

Table 2 shows the results from multivariate linear regression analysis in males and females. In males, multivariate linear regression analysis indicated that age, BMI, blood cadmium, and milk consumption explained 25% of the variability in BMD; none of the organochlorines was significantly associated with BMD when entered into the model individually. The organochlorine variables were then entered stepwise into the model, sequentially, in order of the smallest probability of *F* (if *F* ≤ 0.10), until no more variables were eligible for inclusion or removal (*F* ≥ 0.15). In this model CB-118 was negatively associated (B = −0.00024; *p* = 0.002) and Σ_3PCB_ positively associated (B = 0.00002; *p* = 0.003) with BMD in males, explaining an additional 6% of the variability in BMD. In females, multivariate linear regression analysis indicated that age, BMI, blood cadmium, age at menstruation, and ever having being pregnant explained 39.5% of the variability in BMD. When organochlorines were entered individually into this model, CB-118 was significantly positively associated with BMD (B = 0.00008; *p* = 0.045), explaining a further 2% of the variability in BMD. No additional organochlorines contributed significantly to the model after stepwise entry. Variables representing interactions between the organochlorines and cadmium did not explain any further variability in BMD.

A dichotomous variable representing presence or absence of low BMD (defined as *Z*-score less than −1) was used in logistic regression analyses; 47 of 154 (30.5%) of the males, and 31 of 167 (18.6%) of the females had low BMD. Those organochlorines that were found to significantly explain variability in BMD after stepwise entry into the multivariate linear regression models (CB-118 and Σ_3PCB_ in males and CB-118 in females) were included in the multivariate logistic regression as categorical variables (tertiles). Where a linear dose–response relationship seemed apparent by tertiles, these variables were reentered into the model as continuous variables to maximize power in assessing the dose–response relationship. Table 3 shows the results from logistic regression analysis in males and females. In males, the odds ratio (OR) for low BMD decreased with increasing BMI and milk consumption and increased with increasing blood cadmium levels. The risk of low BMD increased by tertile of CB-118, and when analyzed as a continuous variable, the OR was 1.06 (95% confidence interval 1.01–1.12) for every 10 pg/mL CB-118. The risk of low BMD did not show a clear trend across tertiles of Σ_3PCB_. In women, the ORs for low BMD decreased with increasing BMI and increased with increasing age at menstruation; however, the risk of low BMD did not show a consistent trend with tertiles of CB-118.

## Discussion

The present study is one of the largest to date to assess the relationship between organochlorine levels in blood and BMD in an environmentally exposed population. The findings of this study suggest that even at relatively low levels of organochlorine exposure, BMD might be affected, at least in males, after controlling for major confounding variables. Despite the large variability of the individual values, spanning over three orders of magnitude, the blood concentration levels of these pollutants in this population are in the range of the values detected in human serum and/or blood samples for the general population (Botella et al. 2004; Turci et al. 2004; Wilhelm et al. 2003). Nonetheless, there was a significant positive dose–response relationship in men between the dioxin-like congener CB-118 and BMD (OR = 1.06 per 10 pg/mL; 95% confidence interval 1.01–1.12). In females, after finding a small but significant positive association between CB-118 and BMD in the linear regression analysis, we anticipated a decrease in risk of low BMD with increasing levels of CB-118; however, the risk of low BMD did not show a consistent trend with tertile of CB-118. This contradictory finding, in conjunction with the small change in proportion of BMD variability explained by this variable in the linear regression suggests that CB-118 is unlikely to exert an important influence on BMD in females in this population sample.

In this study, women had significantly higher levels of CB congener 118 and *p,p*′-DDE than did men. Consistently higher levels of CB-118 in women were also reported in Native Americans in the United States (Schaeffer et al. 2006), but higher levels of *p,p*′-DDE in women have not been reported elsewhere. In fact, other studies have reported no difference in *p,p*′-DDE levels by sex (Sandanger et al. 2006) or reported higher *p,p*′-DDE levels in males than in females (Bjerregaard et al. 2001; Jonsson et al. 2005). Lower levels of *p,p*′-DDE in younger females are likely attributable to breast-feeding (which may reduce plasma levels of persistent organic pollutants) and lower dietary exposure; as such, this sex difference is likely to be less evident in populations > 40 years of age, by which age the sex imbalance in organochlorine accumulation due to breast-feeding will be reduced (Sandanger et al. 2003).

These sex differences in organochlorine levels are unlikely to be explained by age, which was similar for the males and females in this study. The main source of exposure to organochlorines for this population is anticipated to be dietary, mainly via fish, to some extent caused by intake of salmon from the Emån river (a well-known salmon-fishing river), which has been polluted with PCBs from an upstream paper mill. More important, the local population may be exposed to PCBs by eating fish from the nearby Baltic Sea. Farmed salmon and wild herring in the Baltic Sea are known to have high concentrations of PCBs. Although individual-level data on fish consumption were not available for analysis, it has been reported by the Swedish National Food Administration that older women eat more fish than older men (Becker et al. 2007). The difference observed in levels of organochlorines in this study could therefore be attributable to a combination of factors including sex-specific differences in consumption patterns, differential metabolism (Landi et al. 1998), or elimination by lactation (Sandanger et al. 2003), although these mechanisms have not been shown to be specific to CB-118 or to *p,p*′-DDE.

No other epidemiologic studies have reported similar sex differences with respect to BMD to those found in this study, although most studies on this topic have focussed on single-sex populations. Similarly the literature on organochlorine exposure and sex-specific effects on BMD in experimental animals is very limited (van der Ven et al. 2006), although sex differences with respect to other outcomes have been observed, possibly indicating a greater sensitivity of males to reproductive system and neurobehavorial effects (Gray et al. 1997a, 1997b). Whether the likely hormonal action of organochlorines could explain or contribute to the different effects on BMD seen in males and females in this study is not clear.

The findings from this study suggest an effect of organochlorine exposure on BMD at levels of exposure experienced in a population in southern Sweden, but there are several limitations of the study that need to be considered. Although this is one of the largest studies to date, when males and females were split into separate groups for analysis, the power of the study to detect statistically significant relationships between organochlorine exposure and BMD was limited. In this study we had data on blood levels of organochlorines in adults, but we do not know about early life exposures, which may be more important than adult exposures in determining the effects of organochlorines on bone. Although it was possible to control for several potentially confounding variables, the linear regression analyses indicate that more than half the variability in BMD is likely to be explained by other, unknown variables that could not be taken into account in this study. Further work should be directed toward establishing whether the sex-specific effects observed in this study are also evident in other environmentally exposed populations. More detailed lifestyle data could be used to elucidate whether any sex-specific effects are caused by a hormonal action of the organochlorines (or the hormonal status of the individuals at the time of exposure), or whether these observations are due to other sex-specific differences related to lifestyle, diet, or occupation.

The purpose of this study was to investigate the relationship between environmental organochlorine exposure and BMD in a population potentially exposed to PCBs from the environment to add to the sparse and inconsistent literature on this important topic. The findings of this study indicate that exposure to some CB congeners, even at relatively low levels, may influence BMD and that this effect may be sex specific.

## Figures and Tables

**Table 1:** Characteristics of study participants, including mean, minimum, and maximum blood levels of measured organochlorines, and percent of samples below the LOD.

			Males (*n* = 154)		Females (*n* = 167)	
Continuous variables			Mean	Range	Mean	Range
Age			70.8	60–81	69.9	60–81
BMI			26.2	18.1–34.5	27.0	17.5–46.3
Blood cadmium (nmol/L)			10.4*	1.0–84.6	5.4*	1.0–45.9
Alcohol (g/week)			10.1*	0–181	2.9*	0–44
Milk (dL/week)			36.1*	0–140	26.7*	0–70
Age at menstruation (*n* = 135)			—	—	13.9	10–18
Age at menopause (*n* = 146)			—	—	48.8	30–85
Reproductive years (*n* = 123)			—	—	35.1	15–75
BMD (g/cm^2^)			0.51*	0.27–0.71	0.38*	0.13–0.64
Dichotomous variables			Yes (%)	No (%)	Yes (%)	No (%)

Low BMD (*Z*-score less than −1)			47 (30.5)	107 (69.5)	31 (18.6)	136 (81.4)
Cortisone (> 3 months)			18 (12)	132 (88)	16 (9.6)	150 (90.4)
Ever pregnant			—	—	142 (85.5)	24 (14.5)
Contraceptive pill use (*n* = 156)			—	—	26 (16.7)	130 (86.3)
Organochlorines (pg/mL)	LOD	% < LOD	Mean	Range	Mean	Range

CB-105	4	7.5	42.1	< LOD–421	49.0	< LOD–366
CB-118	5	0.3	184*	< LOD–1,359	236*	< LOD–1,145
CB-156	8	2.8	116*	< LOD–450	98.5*	< LOD–243
CB-157	3	26	20.0	< LOD–211	16.4	< LOD–64.4
CB-167	2	12	31.6	< LOD–172	34.3	< LOD–127
TEQ_mono-_*_ortho_*	—	—	0.012	0.002–0.067	0.0130	0.003–0.053
CB-138	6	0.0	561	34.0–2,239	582	121–1,461
CB-153	6	0.0	1,290	196–4,360	1,260	328–4,587
CB-180	13.5	0.3	819*	117–3,309	706^*^	< LOD–1,966
Σ_3PCB_	—	—	2,670	438–8,957	2,548	717–7,010
*p,p*′-DDE	8	0.0	2,405*	16–14,268	3,126*	259–17,519

* Significant difference between males and females (*p* < 0.05).

**Table 2:** Multivariate linear regression analyses showing associations between BMD (g/cm^2^) and explanatory variables.

	Males^a^			Females^b^		
Variable	Coefficient (B)	SE	*p*-Value	Coefficient (B)	SE	*p*-Value
Age	−0.004009	0.001123	< 0.001	−0.005635	0.000953	< 0.001
BMI	0.008255	0.002006	< 0.001	0.006300	0.001209	< 0.001
ln blood-Cd (nmol/L)	−0.013383	0.005946	0.026	−0.010184	0.008257	0.220
Milk (dL/week)	0.000544	0.000245	0.028	—	—	—
Age at menstruation	—	—	—	−0.009020	0.003598	0.013
Ever pregnant	—	—	—	0.037579	0.015894	0.020
CB-118	−0.000110	0.000062	0.079	0.000080	0.000039	0.045
TEQ_mono-_*_ortho_*	−0.225387	1.156013	0.846	1.652927	0.861054	0.057
CB-153	0.000011	0.000011	0.331	0.000009	0.000010	0.385
Σ_3PCB_	0.000008	0.000006	0.154	0.000007	0.000006	0.223
*p,p*′-DDE	−0.000003	0.000003	0.323	0.000003	0.000002	0.184

Age, BMI, blood cadmium, and milk (males) or age at menstruation and ever pregnant (females) entered simultaneously; organochlorines then entered individually into the model.

a Adjusted for age, BMI, blood-cadmium, and milk consumption (final sample size = 150).

b Adjusted for age, BMI, blood-cadmium, age at menstruation, and ever pregnant (final sample size = 134).

**Table 3:** Logistic regression model for low BMD (*Z*-score less than −1) in males and females.

Variable	OR (95% CI)	*p*-Value
Males (*n* = 150)^a^		
BMI	0.83 (0.72–0.95)	0.009
ln Blood cadmium (nmol/L)	1.76 (1.19–2.59)	0.004
Milk consumption (dL/week)	0.97 (0.95–0.99)	0.004
CB-118 (< 33rd percentile)	1.00 (referent)	—
CB-118 (33rd–67th percentile)	1.48 (0.55–3.97)	0.437
CB-118 (> 67th percentile)	2.11 (0.63–7.12)	0.227
CB-118 (continuous variable)	1.06 (1.01–1.12)	0.027
Σ_3PCB_ (< 33rd percentile)	1.00 (referent)	—
Σ_3PCB_ (33rd–67th percentile)	1.17 (0.44–3.16)	0.753
Σ_3PCB_ (> 67th percentile)	0.91 (0.27–3.02)	0.879
Σ_3PCB_ (continuous variable)	1.00 (0.99–1.00)	0.101
Females (*n* = 134)^a^		
BMI	0.80 (0.70–0.93)	0.003
ln Blood cadmium (nmol/L)	0.88 (0.40–1.92)	0.745
Age at menstruation	1.48 (1.07–2.03)	0.016
Ever pregnant	0.47 (0.13–1.70)	0.251
CB-118 (< 33rd percentile)	1.00 (referent)	—
CB-118 (33rd–67th percentile)	2.29 (0.66–7.92)	0.191
CB-118 (> 67th percentile)	2.13 (0.58–7.84)	0.255

CI, confidence interval. In males, BMI, blood cadmium, milk, CB-118 (tertiles) and Σ_3PCB_ (tertiles) were entered simultaneously; tertiles of CB-118 and Σ_3PCB_ were then replaced with the continuous variables, also entered simultaneously. In females, BMI, blood cadmium, age at menstruation, ever pregnant, and CB-118 (tertiles) were entered simultaneously.

a Final sample size.
